# Prey Lysate Enhances Growth and Toxin Production in an Isolate of *Dinophysis acuminata*

**DOI:** 10.3390/toxins11010057

**Published:** 2019-01-21

**Authors:** Han Gao, Mengmeng Tong, Xinlong An, Juliette L. Smith

**Affiliations:** 1 Virginia Institute of Marine Science, College of William & Mary, Gloucester Point, VA 23062, USA; gghanbing@zju.edu.cn (H.G.); jlsmith@vims.edu (J.L.S.); 2 Ocean College, Zhejiang University, Zhoushan 316000, China; 3 Ocean College, Agricultural University of Hebei, Qinhuangdao 066000, China; axlqhd@126.com

**Keywords:** *Dinophysis acuminata*, *Mesodinium rubrum*, lysate, organic matter, diarrhetic shellfish poisoning, okadaic acid, dinophysistoxin, pectenotoxins

## Abstract

The physiological and toxicological characteristics of *Dinophysis acuminata* have been increasingly studied in an attempt to better understand and predict diarrhetic shellfish poisoning (DSP) events worldwide. Recent work has identified prey quantity, organic nitrogen, and ammonium as likely contributors to increased *Dinophysis* growth rates and/or toxicity. Further research is now needed to better understand the interplay between these factors, for example, how inorganic and organic compounds interact with prey and a variety of *Dinophysis* species and/or strains. In this study, the exudate of ciliate prey and cryptophytes were investigated for an ability to support *D. acuminata* growth and toxin production in the presence and absence of prey, i.e., during mixotrophic and phototrophic growth respectively. A series of culturing experiments demonstrated that the addition of ciliate lysate led to faster dinoflagellate growth rates (0.25 ± 0.002/d) in predator-prey co-incubations than in treatments containing (1) similar levels of prey but without lysate (0.21 ± 0.003/d), (2) ciliate lysate but no live prey (0.12 ± 0.004/d), or (3) monocultures of *D. acuminata* without ciliate lysate or live prey (0.01 ± 0.007/d). The addition of ciliate lysate to co-incubations also resulted in maximum toxin quotas and extracellular concentrations of okadaic acid (OA, 0.11 ± 0.01 pg/cell; 1.37 ± 0.10 ng/mL) and dinophysistoxin-1 (DTX1, 0.20 ± 0.02 pg/cell; 1.27 ± 0.10 ng/mL), and significantly greater total DSP toxin concentrations (intracellular + extracellular). Pectenotoxin-2 values, intracellular or extracellular, did not show a clear trend across the treatments. The addition of cryptophyte lysate or whole cells, however, did not support dinoflagellate cell division. Together these data demonstrate that while certain growth was observed when only lysate was added, the benefits to *Dinophysis* were maximized when ciliate lysate was added with the ciliate inoculum (i.e., during mixotrophic growth). Extrapolating to the field, these culturing studies suggest that the presence of ciliate exudate during co-occurring dinoflagellate-ciliate blooms may indirectly and directly exacerbate *D. acuminata* abundance and toxigenicity. More research is required, however, to understand what direct or indirect mechanisms control the predator-prey dynamic and what component(s) of ciliate lysate are being utilized by the dinoflagellate or other organisms (e.g., ciliate or bacteria) in the culture if predictive capabilities are to be developed and management strategies created.

## 1. Introduction

The dinoflagellate *Dinophysis* spp. has been associated with diarrhetic shellfish poisoning (DSP) events worldwide due to human exposure to the toxin okadaic acid (OA) and its derivatives, dinophysistoxins (DTXs) [1,2]. These lipophilic compounds can accumulate in filter-feeding bivalves and adversely affect humans and other animal consumers. As strong inhibitors of serine and threonine protein phosphatases, DSP toxins can promote potent tumors [3], induce intestinal distress such as vomiting and diarrhea [2,4], and limit the growth of phytoplankton competitors [5,6]. Many toxigenic *Dinophysis* spp. also synthesize pectenotoxins (PTXs), a class of bioactive, polyether lactones. While not a contributor to DSP, some pectenotoxins are acutely toxic to vertebrate models via intraperitoneal injection [7,8], and therefore, the toxin class is regulated in the European Union [9].

With the threat of DSP appearing to be on a rise globally and emerging in new regions, e.g., U.S. coastlines, investigations into the possible drivers of *Dinophysis* spp. growth and toxin production have become a growing area of research in the last decade. This important work was made possible through the revolutionary discovery by Park et al. [10]: to grow in culture, *Dinophysis* spp. must be fed ciliates, *Mesodinium rubrum*, that previously grazed upon cryptophytes of the *Teleaulax/Geminigera* clade [11]. Molecular evidence supports the need for this multi-stage culturing scheme, as *Dinophysis* and *Mesodinium* plastids have been shown to originate from cryptophytes [12,13,14,15,16] and *Dinophysis* cells have been found in the field to concurrently contain plastids originating from different strains of cryptophyte [17]. More specifically, ciliates of the genus *Mesodinium* capture, sequester, and regulate the nuclear genome of its cryptophyte prey [11,15,16], after which, *Dinophysis* consumes the plastids in the ciliates by kleptoplasty via a peduncle. Stemming from this advancement in culturing, numerous *D. acuminata* isolates [10,18,19,20,21,22,23,24,25,26] and isolates of other *Dinophysis* spp. [21,27,28,29,30,31] have been successfully established, allowing now for comparisons between geographical strains and species.

Prior to the multi-stage culturing technique put forth by Park et al. [10], however, multiple types of organic material, in both dissolved and particulate form, were trialed in an attempt to culture *Dinophysis* as a monoculture, including dissolved organic materials (soil extract, humic acid, dextrans, urea, glutamic acid, hypoxanthine, gibberellic acid, indol acetic acid, kinetin, polyamines, lectins of *Phaseolus* and porcine blood platelets) [32], and live prey (bacteria, pico- and nanoplankton, and yeast) [33]. None of these trials with organic materials supported *Dinophysis* spp. growth enough to allow for successful isolation of the genera and the establishment of cultures, leading to the assumption that *Dinophysis* could not directly utilize organic compounds.

Recent studies with isolates, however, demonstrate that a variety of organic materials, and some inorganics, may benefit *Dinophysis* by indirectly or directly supporting growth and/or toxin production. Toxin production, but not growth, increased when a non-axenic monoculture of *D. acuminata* was supplemented with lysed ciliates and cell debris during a preliminary study [21], and *D. acuminata* growth in monocultures and predator-prey co-incubations was enhanced with the addition of urea, glutamine, or waste water organic matter [24]. With respect to inorganic nutrients, three recent studies have confirmed that *D. acuminata* does not readily utilize nitrate to support growth [24,34,35] or toxin production [34,35], but that the ciliate prey rapidly assimilates nitrate to promote its own division, thereby indirectly supporting *D. acuminata* [35]. Ammonium, interestingly, was shown to likely play a direct role in *D. acuminata* growth, bloom development, and toxin production, through uptake of this inorganic compound by the dinoflagellate [24,34,36,37]. What’s improved in these later culturing studies, as compared to Nagai et al. [21], is that compounds of interest were added to *Dinophysis* with and without prey as a food source, examining therefore, growth and toxin production during mixotrophic and phototrophic growth.

By including prey in a treatment with possible nutrient sources, additional questions can be asked regarding the combined roles of ciliates and nutrients in dinoflagellate growth and toxin production. More specifically, a pairing of prey cells and prey exudate or lysate seems more environmentally relevant than testing in solidarity, as the two are likely found in conjunction within a system. Co-occurrence of cells and released internal components, for example, may occur throughout a bloom, due to such processes as sloppy feeding or cell division; however, the presence of these compounds likely increases in the surrounding waters near the termination of a ciliate bloom when cells may be experiencing aging and membrane permeability, parasitic lysis, or cell death. This progression has been demonstrated in the laboratory for endotoxins OA and DTX1 [21,38]. Additionally, ciliates should be considered in this relationship as laboratory studies [10,39,40,41] have indicated prey abundance as an important controller of *Dinophysis* growth and/or toxin production, and *Dinophysis* spp. have been found to bloom immediately after and co-occur with ciliate prey in the field [42], suggesting that factors controlling ciliate abundance and distribution are important to down-stream DSP events. Further research is now needed to further understand how inorganic and organic compounds interact with prey and a variety of *Dinophysis* spp. and isolates if predictive capabilities are to be developed and management strategies created.

Building on a previous study [21], mixotrophic *D. acuminata* was investigated for its ability to utilize organic material released from the ciliate, *M. rubrum* in the presence or absence of the ciliate as a food source, i.e., during mixotrophic vs. phototrophic growth, respectively. We supplemented a *D. acuminata* culture, comprising of f/6-Si medium [43], with ciliate lysate (derived from probe-sonification of ciliate culture), ciliate lysate and live ciliates, or live ciliates alone at two initial cell concentrations (Table 1). To determine if any measured effect on *D. acuminata* growth or toxin production was unique to the ciliate lysate, we also amended *D. acuminata* culture with cryptophyte lysate or live cryptophytes. All treatments were compared to a monoculture control of *D. acuminata* with no organic amendments, and cultures of *D. acuminata* were starved, in the light, for two weeks before the experiment to ensure that any responses measured in the dinoflagellate were due to the amendments, and not sustained growth or divisions using internal reserves. While the main objective of this study was to begin investigating, in the laboratory, if organic matter derived from a co-occurring bloom of ciliates could support *Dinophysis* growth and toxin production either directly or indirectly, the information may also have implications for future *D. acuminata* isolation attempts. If lysate, for example, promotes *D. acuminata* growth, then this may be a mechanism to increase the likelihood of isolation success.

## 2. Results

### 2.1. Lysate Size Characterization

Particles (*n* = 185) in the ciliate lysate were photographed and measured under a light microscope at 100×. The mean size of lysate particles was 3.41 ± 0.13 μm (mean ± standard error) in diameter and 9.17 ± 0.73 μm^2^ in area. Attempts were also made to characterize particles in the cryptophyte lysate; however, particles were not large enough to quantify using supplied magnification and software.

### 2.2. Growth of Dinoflagellate, Ciliate, and Cryptophyte

**Figure 1:**
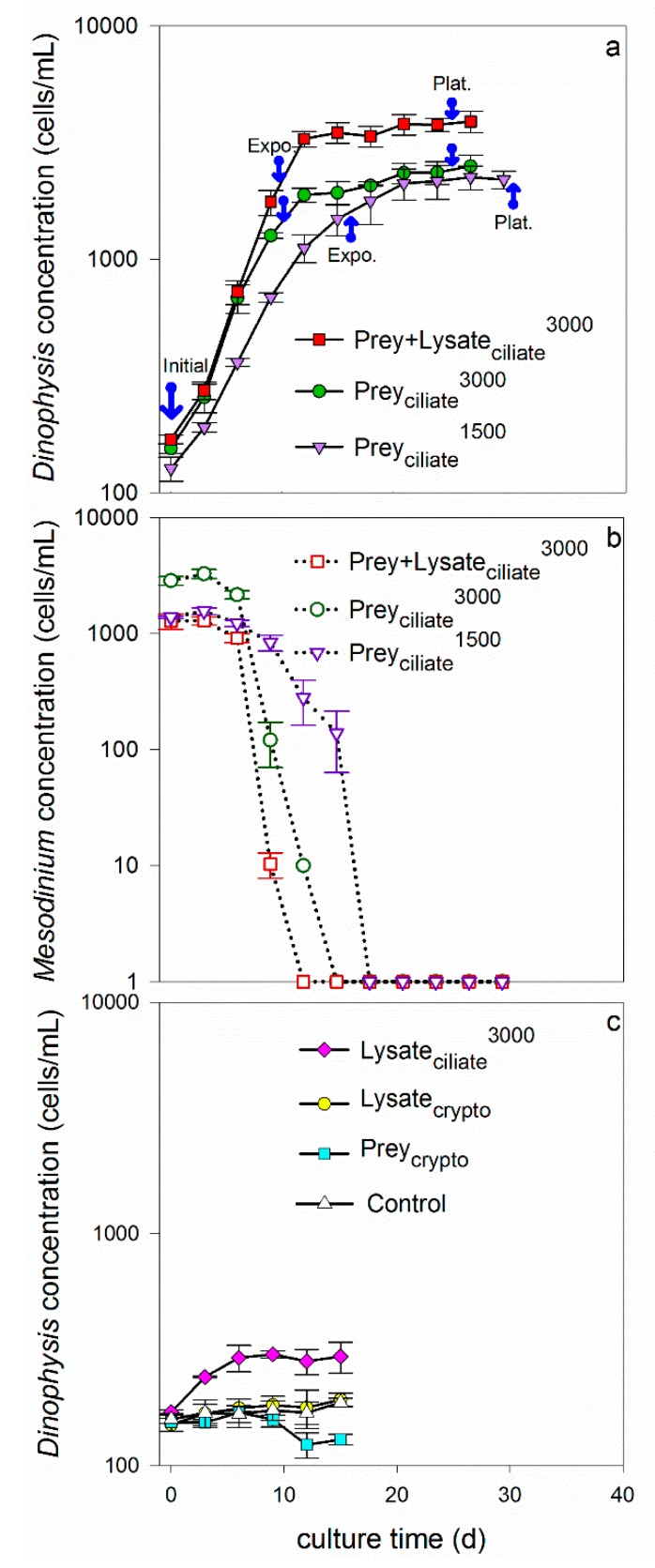
Growth response of *Dinophysis acuminata* (**a**,**c**) and ciliates, *M. rubrum* (**b**), in different treatments after being starved, in the light, for two weeks before the experiment to ensure the prey were fully consumed from the medium and that any responses measured in *Dinophysis* were due to the amendments and not sustained growth or divisions using internal reserves. Treatments include Prey_ciliate_^3000^: with ciliates at 3000 cells/mL; Prey + Lysate_ciliate_^3000^: with ciliates at 1500 cells/mL + ciliate lysate equivalent to 1500 cells/mL; Prey_ciliate_^1500^: with ciliates at 1500 cells/mL (**a**,**b**); Lysate_ciliate_^3000^: with ciliate lysate equivalent to 3000 cells/mL; Lysate_crypto_: with cryptophyte lysate equivalent to 15,000 cells/mL; Prey_crypto_: with cryptophytes at 15,000 cells/mL; and Control: with no prey or lysate addition (**c**). Initial concentration of *D. acuminata* was 150 cells/mL for all treatments. Mean values and standard deviations are plotted (*n* = 3). Blue arrows in (**a**) indicate when samples were initially harvested for toxin analysis, and then during exponential (Expo.) and plateau (Plat.) growth phases.

Dinoflagellates grew exponentially in the four treatments that were fed live ciliates (1500 or 3000 cells/mL), a 1:1 mixture of living ciliates and lysate (equivalent to 3000 cells/mL), and only ciliate lysate (equivalent to 3000 cells/mL). More specifically, growth rates were 0.16 ± 0.007, 0.21 ± 0.003, 0.25 ± 0.002, and 0.12 ± 0.004/d for treatments Prey_ciliate_^1500^, Prey_ciliate_^3000^, Prey + Lysate_ciliate_^3000^, and Lysate_ciliate_^3000^, respectively (Table 1, Figure 1). When compared across treatments providing the same concentration of ciliates, 3000 cell eq./mL, *Dinophysis* grew faster when provided prey or a combination of prey and lysate (treatments Prey_ciliate_^3000^ and Prey + Lysate_ciliate_^3000^), than when provided only lysate (Lysate_ciliate_^3000^) (*t*-test, *p* < 0.05). Similarly, when provided both ciliate prey and lysate (Prey + Lysate_ciliate_^3000^), *Dinophysis* reached a higher maximum, final biomass (3900 cell/mL) at plateau phase than when it was provided with only living ciliates (Prey_ciliate_^3000^), 2500 cells/mL, or only lysate (Lysate_ciliate_^3000^), 302 cells/mL (Table 1, Figure 1). The average maximum biomass of *Dinophysis* in the Lysate_ciliate_^3000^ treatment was significantly greater than the maximum biomass measured in the monoculture control, showing that growth was supported for three days on materials liberated from the ciliate lysate (after two weeks of starvation in the light). When comparing between treatments providing live ciliates, *Dinophysis* grew significantly faster during exponential growth in the 3000 cells/mL treatment (Prey_ciliate_^3000^) than in the 1500 cells/mL treatment (Prey_ciliate_^1500^); however, *Dinophysis* in the latter treatment grew steadily for a longer period, resulting in no detectable difference in maximum biomass between treatments (Table 1). The dinoflagellates in treatments that were provided with either living cryptophytes (Prey_crypto_), cryptophyte lysate (Lysate_crypto_), or no additions (Control, growth rate = 0.01 ± 0.007/d) did not show evidence of exponential growth over the experimental period (Table 1, Figure 1).

The cell concentrations of ciliates and cryptophytes were also monitored in the co-incubations. The cell concentration of the ciliate quickly decreased over time as *Dinophysis* fed during exponential growth. More specifically, ciliates were completely consumed by days 15–18 in the two treatments to which they were added without lysate: Prey_ciliate_^1500^ and Prey_ciliate_^3000^ (Figure 1). The prey in the Prey + Lysate_ciliate_^3000^ treatment were depleted from the co-incubation by day 12, however, transitioning *Dinophysis* into a prey-limited phase earlier than the other two treatments with live ciliates. The cryptophytes that were co-incubated with *Dinophysis* (Prey_crypto_) grew exponentially for 12 days, reaching a maximum concentration of 600,000 cells/mL during the experimental period (data not shown).

### 2.3. Toxin Quota, Concentration, and Production

Cells and media of *Dinophysis* were harvested separately and analyzed for toxin during two growth phases, exponential and plateau, for three treatments: Prey_ciliate_^1500^, Prey_ciliate_^3000^, and Prey + Lysate_ciliate_^3000^. Initial toxin samples were also collected and analyzed from the inoculum *Dinophysis* cultures for comparison. Toxin data are not reported for the other four treatments, because growth rates and final biomass were significantly lower when *Dinophysis* was grown with only ciliate lysate, grown with cryptophytes that were living or lysed, or grown as a monoculture control. More specifically, *Dinophysis* in these latter treatments (1) produced insufficient biomass to reach toxin detection limits, and/or (2) did not progress through typical growth phases, i.e., lag, exponential, and plateau, during the experimental period, making the toxin data incomparable to the high-growth treatments.

We calculated the potential loss of *D. acuminata* during the harvesting process for toxin samples, i.e., sieving. Only 1% of the harvested cells were potentially lost during harvesting; possible mechanisms include being trapped in/on the sieve, loss of small cells, and/or artificial cell lysis during processing. This error was found to be minimal and constant over growth phases, and therefore, considered irrelevant to this study.

**Figure 2:**
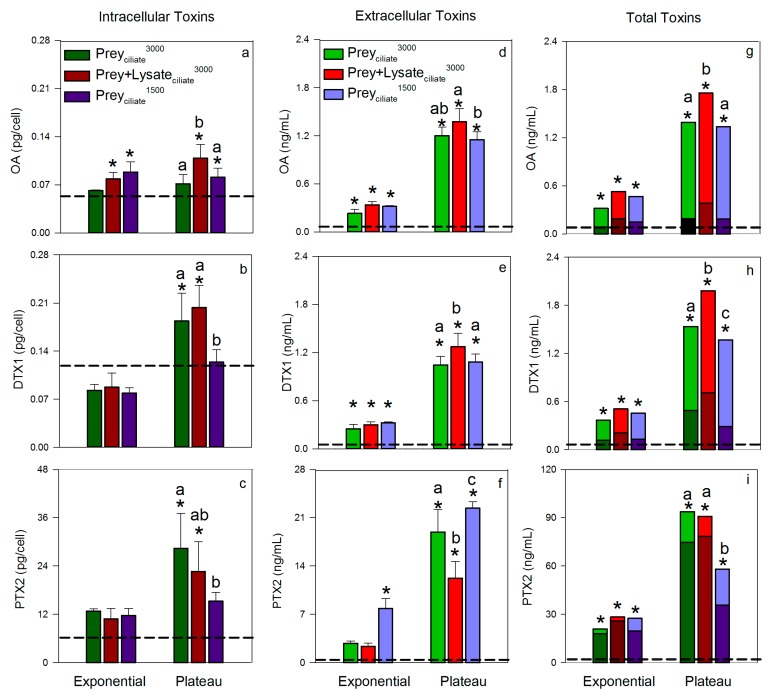
Toxin levels in *Dinophysis acuminata* cells and medium over two growth phases: exponential and plateau for three treatments. (**a**–**c**, Mean ± SD, *n* = 3) intracellular toxin quotas, (**d**–**f**, Mean ± SD, *n* = 3) extracellular toxin concentrations in the cultures, and (**g**–**i**) total toxin concentration in *D. acuminata* cultures with ciliate prey and/or lysate (Mean, *n* = 3). Intracellular toxins are indicated with darker color bars, while extracellular toxins are represented using the lighter color bars. Toxins quantified include okadaic acid (OA), dinophysistoxin-1 (DTX1), and pectenotoxin-2 (PTX2). Dashed horizontal line in each panel indicates the mean initial toxin level at the start of the experimental period. Asterisks ‘*’ indicate that a treatment was significantly greater at that growth phase as compared to the initial toxin level, and letters that are uncommon show significant difference (*p* < 0.05) in toxin levels between treatments during plateau phase, within that respective panel only.

Toxin quotas (intracellular toxin) and concentrations (extracellular toxins) measured during exponential and plateau phases were compared to the initial toxin levels in the maintenance *Dinophysis* culture that was used for inoculation (taken in late plateau phase) to demonstrate increases in toxin relative to initial conditions. As a general trend, intracellular levels of OA, DTX1, and PTX2 remained minimal, i.e., similar to initial levels, during exponential growth, but increased during plateau phase (Figure 2a–c). Exceptions were observed, however, whereby *Dinophysis* that was provided ciliate prey at an initial concentration of 3000 cells/mL (Prey_ciliate_^3000^) did not increase their OA toxin quota, relative to initial conditions, over the entire experimental period. Additionally, *Dinophysis* fed the lower initial concentration of ciliate (Prey_ciliate_^1500^) did not increase their internal quotas of DTX1 or PTX2 above initial conditions over the entire experimental period. Maximum average toxin quotas were reached during plateau phase for OA, 0.11 ± 0.01 pg OA/cell (Prey + Lysate_ciliate_^3000^); DTX1, 0.20 ± 0.02 pg/cell (Prey + Lysate_ciliate_^3000^); and PTX2, 28.38 ± 5.02 pg/cell (Prey_ciliate_^3000^).

As seen with intracellular toxin quotas, extracellular toxin concentrations of OA, DTX1, and PTX2 in the culture medium of the three treatments remained low, relative to initial toxin concentrations, through exponential growth, but significantly increased by plateau phase (Figure 2d–f). Maximum, average extracellular toxin concentrations were reached during plateau growth phase for OA, 1.37 ± 0.01 ng/mL (Prey + Lysate_ciliate_^3000^) DTX1, 1.27 ± 0.10 ng/mL (Prey + Lysate_ciliate_^3000^); and PTX2, 22.37 ± 0.54 ng/mL (Prey_ciliate_^1500^). Once the intracellular quotas were converted to intracellular toxin concentrations per milliliter of culture and combined with extracellular toxin concentrations to yield total toxin concentrations (Figure 2g–i), it became apparent that total toxin concentration showed the same overall pattern across treatments; total toxins increased over the aging of the culture and reach maximum levels by plateau phase. DSP toxins, OA and DTX1, were more associated with the extracellular fraction, <15 µm, while PTX2 was associated with the cells and particulates, ≥15 µm.

Total toxin concentrations, toxin quotas, and extracellular concentrations were also compared across treatments during plateau phase to look for any effect from the different organic amendments (Figure 2). Overall, the total toxin concentrations of OA and DTX1 were significantly greater in the Prey + Lysate_ciliate_^3000^ treatment, as compared to the treatments with only live ciliates added (Prey_ciliate_^1500^, Prey_ciliate_^3000^). When evaluating intra and extracellular toxins separately, the same trend remained: the mean OA toxin quota and DTX1 extracellular concentration were significantly greater than treatments with live prey. Okadaic acid extracellular concentrations (Figure 2d) and DTX1 intracellular toxin quotas (Figure 2b), however, were indistinguishable between the two treatments with lysate (Prey + Lysate_ciliate_^3000^) and the higher prey abundance (Prey_ciliate_^3000^). In all cases, Prey + Lysate_ciliate_^3000^ and the higher prey abundance Prey_ciliate_^3000^ contained more OA or DTX1 in the intra or extra cellular fractions than the treatment with the lowest prey abundance (Prey_ciliate_^1500^). Pectenotoxin-2 levels did not follow a distinguishable trend across treatments or toxin measurements, but values were more than a magnitude greater than OA and DTX1 combined.

The rate of intracellular toxin production (OA, DTX, or PTX2) by *Dinophysis* was greater during exponential growth phase than as cells transitioned into plateau phase (Table 2). Toxin production rates of OA, DTX1, and PTX2 were significantly faster (*p* < 0.05) when *Dinophysis* was provided with a mixture of prey and lysate (Prey + Lysate_ciliate_^3000^) than when provided the same concentration of prey, but no lysate (Prey_ciliate_^1500^). Toxin production in the mixed treatment and higher prey treatment (Prey_ciliate_^1500^) were more comparable. Toxin production occurred during the transition from exponential to plateau phase as well; however, rates of OA, DTX1, and PTX2 production were reduced (Table 2).

## 3. Discussion

Mixotrophic *Dinophysis acuminata* was investigated for its ability to utilize organic material released from the ciliate, *Mesodinium rubrum*, or the cryptophyte, *Teleaulax amphioxeia*. As such, an isolate of *D. acuminata* was grown in the presence of live cells, lysate, live cells and lysate, or no amendments except fresh culture medium. Overall, the addition of ciliate lysate, but not cryptophyte lysate, to co-incubations of predator and prey led to significantly higher *Dinophysis* growth rate, biomass, OA toxin quotas, DTX1 extracellular toxin concentrations, and DSP total toxins (Figure 1 and Figure 2, and Table 1 and Table 2). It is important to note, that the addition of ciliate lysate to co-incubations increased growth and toxigenicity above similar treatments where *Dinophysis* was provided with the same or more live ciliate prey (1500 and 3000 cells/mL, respectively) or the same amount of overall ciliate biomass as lysate (3000 cell eq/mL). Together these results demonstrate that the organic matter from the lysed ciliates provided the necessary nutrition to *Dinophysis* (directly or indirectly) to support growth (phototrophic or mixotrophic, respectively) and toxin production by the dinoflagellate.

### 3.1. Ciliate Lysate in Support of Growth

Early culturing experiments proposed that *Dinophysis acuminata* required both prey and light to grow in culture [10,22,39,40,41]; however, more recently, *D. acuminata* has been shown to undergo phototrophic growth, in the presence of ammonium and urea, and the absence of prey, but that growth occurs at a slower rate than during mixotrophy [34,36]. Similarly, in the present study, an isolate of *D. acuminata* from Eel Pond, USA, grew for 3 days and reached significantly higher biomass when provided only ciliate lysate as an amendment beyond medium (Lysate_ciliate_^3000^, Figure 1) as compared to a monoculture control that showed no growth or change in biomass over the same period. These dinoflagellate cultures were starved for two weeks in the light prior to the experiment to ensure that internal reserves were depleted and that any responses measured in *Dinophysis* were due to the new amendments. While certain growth (photosynthetic + mixotrophic) occurred without prey in the ciliate lysate treatment (Lysate_ciliate_^3000^), growth rates and final biomass significantly increased once prey were added to *D. acuminata* culture (Prey + Lysate_ciliate_^3000^, Prey_ciliate_^1500^, Prey_ciliate_^3000^) (Table 1), supporting previous findings for faster growth rates during mixotrophic growth. It is unclear whether the dinoflagellate directly and/or indirectly benefitted from the organic materials liberated from the ciliates. Other studies have proposed an indirect link between dissolved nutrients and *Dinophysis* abundance, citing the ciliate’s high affinity for dissolved inorganic and organic nutrients and a cascading increase in ciliate abundance and then *Dinophysis* abundance [34,35,44]. Ciliates, however, did not increase in abundance or extend their presence in the co-incubation with the addition of lysate (Figure 1b); this is contrary to what can be expected if the ciliate was the link between the liberated organic materials and increased *Dinophysis* abundance. This result, instead, lends support for an alternative conclusion: *D. acuminata* directly used the liberated materials to enhance total (photosynthetic + mixotrophic) growth on top of mixotrophic growth.

The growth-promoting effect of ciliate lysate also surpassed the effects of doubling ciliate abundance and exceeding prey saturation levels. More specifically, doubling the concentration of live ciliates inoculated into co-incubations from 1500 to 3000 cells/mL (Prey_ciliate_^1500^, Prey_ciliate_^3000^, respectively), significantly increased *Dinophysis* growth rate (Figure 1, Table 1). This increase in rate and biomass achieved with more prey, however, was still below that achieved by *D. acuminata* when grown in the presence of lysate and less prey (Prey + Lysate_ciliate_^3000^). Differences in growth rate between the low and high prey treatments were expected given that the initial prey concentrations, 1500 and 3000 cells/mL respectively, were below and above the published prey saturation threshold: ~2000 cells/mL [39,45]. The design of these treatment levels was intentional in that it allowed for the examination of whether a sub-saturation level of live prey, as seen in treatments Prey + Lysate_ciliate_^3000^ and Prey_ciliate_^1500^, would lead to similar maximum *Dinophysis* growth rates and biomass, or if supplementing that sub-saturation level of prey with lysate would allow *D. acuminata* to perform at the level of super-saturation, i.e., equivalent to the high prey treatment, Prey_ciliate_^3000^. As the latter outcome was observed, i.e., *D. acuminata* grew fastest in the lysate and prey treatment (Prey + Lysate_ciliate_^3000^), one can conclude that either (1) the ciliate (or associated bacteria) utilized the liberated materials promoting *D. acuminata* growth by making itself more enriched or abundant, or (2) with the addition of ciliate lysate, total (photosynthetic + mixotrophic) growth was enhanced in *D. acuminata* (i.e., direct uptake of liberated materials by dinoflagellate).

Alternatively, the bacteria associated with the ciliate culture inoculum may be indirectly responsible for the measured increase in *D. acuminata* growth in the mixed treatment (Prey + Lysate_ciliate_^3000^). The three cultures of cryptophyte, ciliate, and dinoflagellate used in this study were non-axenic, and so likely contained heterotrophic bacteria at the time of co-incubation. Bacteria, in general, are well known for their contributions to phytoplankton growth [46,47,48], and may have remineralized the organic material (dissolved or particulate) into nutrient chemical forms that *D. acuminata* can directly utilize to support cell division, such as ammonium or urea [34,49]. Growth factors, such as essential vitamins and metals, may also be liberated through remineralization of dissolved organic substrates. Therefore, interactions between *Dinophysis*-ciliate-bacteria still need to be evaluated systematically if the link between the dinoflagellate, ciliate, and/or organic compounds or materials is to be realized.

As ciliate lysate was shown to enhance *D. acuminata* growth in the presence of ciliate inoculum (i.e., live ciliates and associated bacteria), then ciliate lysate addition may be a mechanism to increase the likelihood of success during *D. acuminata* isolation. While early attempts to isolate and establish *Dinophysis* cultures with organic matter failed [32,33], more recent studies with field populations and established isolates suggest *Dinophysis* experiences direct or indirect benefits from organic compounds [21,24,34,36] and this study. Given how critical the multi-stage feeding scheme [10] is to the successful isolation of *Dinophysis* spp., we do not recommend replacing prey with ciliate lysate, but instead suggest that in addition to typical *Dinophysis* isolation methods, researchers consider adding equal parts of live ciliates and ciliate lysate to a subset of single-cells in a well plate. Note that the growth-promoting effect of ciliate lysate has only been demonstrated for *D. acuminata* and so it is not known if these results extrapolate to others in the genus. Additionally, ciliates used in this study were maintained by performing a 1:2 dilution with fresh medium (see Materials and Methods), meaning that ciliates were likely conditioned to utilize additional nutrient forms than those that are strictly provided in f/6-Si medium. It is, therefore, unknown if the growth-promoting effect of ciliate lysate, in the present study, was dependent upon the pre-conditioning of ciliates, and therefore *D. acuminata*, to utilize multiple chemical forms.

### 3.2. Ciliate Lysate in Support of Toxin Production

The addition of a mixture of ciliate lysate and live ciliates to *D. acuminata* cultures significantly increased total toxin concentrations (intracellular + extracellular) of OA and DTX1 relative to both initial levels and the other treatments that provided only ciliates (Figure 2g–h). Pectenotoxin-2 did not show a consistent trend across treatments and toxin fractions, but did significantly increase in the lysate and prey treatment from initial levels (Figure 2c,f,i). These novel findings built upon a previous study by Nagai et al., [21], whereby the authors showed that the addition of lysate alone increased total toxin levels in a monoculture of *D. acuminata*. While the current study cannot ask the same question, i.e., the lysate-only treatment did not produce enough biomass for toxin testing, it is interesting that both studies reported a toxin-promoting effect of lysate. The consistency in results between studies, two geographical isolates, and different prey lines, lends support to this result’s validity. Additionally, the earlier depletion of live ciliates from the Prey + Lysate_ciliate_^3000^ treatment (Figure 1b) may have contributed to the enhanced toxin quotas in *D. acuminata* as toxin content has been shown to increase in cultures during the prey-limited phase (52).

Okadaic acid and DTX1 released into the surrounding environment, whether actively or passively, by *D. acuminata* may have detrimental effects on competitors and prey. These toxins have been shown to limit the growth of several microalgae [5,6] and have been proposed to aid in the capture and immobilization of ciliate prey [31,40,50,51]. Ciliates, *M. rubrum*, exhibited abnormal behavior when exposed to high densities of *D. fortii*, such as forming clumps or rotating at the same place, followed by death [51]. Other mechanisms of capture have also been investigated, such as mucus traps [30] and chemical or physical sensing [52,53], in which OA has either been found unimportant to the mechanism or not evaluated as a contributor. The present study suggests a positive feedback may occur, whereby the addition of prey lysate enhances the production and exudation of DSP toxins. The addition of ciliate lysate to co-incubations (Prey + Lysate_ciliate_^3000^) resulted in a significant increase in OA and DTX1 levels to 0.38 and 0.71 ng/mL intracellularly, and 1.37 and 1.27 ng/mL extracellularly by plateau phase, respectively (Figure 2g–h). Mechanisms underlying this feedback, e.g., chemical detection of prey [52], should be further explored as these data suggest that the presence of cells and/or their exudate will enhance extracellular DSP toxin levels.

## 4. Conclusions

To summarize, an isolate of *D. acuminata* benefitted from the addition of organic matter that was released from *M. rubrum* by probe-sonification. Growth and DSP toxin metrics elevated when the lysate was administered with live prey, suggesting that either total (photosynthetic + mixotrophic) growth of *D. acuminata* was enhanced with the addition of the liberated organic compounds and/or the ciliate (or associated bacteria) was directly utilizing the liberated materials and indirectly providing the benefit to *D. acuminata* through remineralization to bioavailable forms or increasing ciliate enrichment and/or abundance. When provided with ciliate lysate only, however, growth of *D. acuminata* was also observed, lending some support to the conclusion that the dinoflagellate does have some capacity to directly utilize the liberated compounds itself and promote certain growth and toxin production.

Extrapolating to the field, these culturing studies suggest that the co-occurrence of a ciliate-dinoflagellate bloom may exacerbate *D. acuminata* abundance and toxigenicity due to exudate or lysing. The lysate from the cryptophyte, however, did not support *Dinophysis* growth, suggesting this growth-promoting effect may be unique to ciliates. Further studies should be conducted to better understand the interactions between organic compounds and *Dinophysis*-ciliate-bacteria relationships and characterize the nutritional composition of ciliate lysate to identify the beneficial components as those might be important drivers of *D. acuminata* abundance and DSP events in the field.

## 5. Materials and Methods

### 5.1. Culture Maintenance

A uni-algal culture of *Dinophysis acuminata* (DAEP01) was previously isolated from Eel Pond, Woods Hole, MA, in September of 2006 [54]. The ciliate *Mesodinium rubrum* (GenBank accession NO. AB364286) and cryptophyte *Teleaulax amphioxeia* (GenBank accession NO. AB364287) were isolated from Inokushi Bay in Oita Prefecture, Japan, in February of 2007 as described in [27]. All cultures were maintained at a salinity of 30 in f/6-Si medium, which was prepared with 1/3 nitrate, 1/3 phosphate, 1/3 metals, and 1/3 vitamins of f/2-Si medium. The three-stage culture system [10] was utilized; the cryptophyte was fed to the ciliate prior to being fed to the dinoflagellate. In detail, every 7 days, 20 mL of *M. rubrum* culture medium was added to 20 mL of fresh f/6-Si medium with 0.5 mL *T. amphioxeia* cultures, providing roughly a 1:2 dilution of ciliate culture. After the cryptophyte cells were consumed, the ciliates were then fed to *Dinophysis* cells at a prey: predator ratio of 3:1. Cultures were maintained at 15 °C with dim light (ca. 20 µmol photons m^−2^ s^−1^) on a 14 h light: 10 h dark photo cycle.

### 5.2. Lysate Preparation and Size Characterization

*Mesodinium rubrum* and *T. amphioxeia* cultures in early plateau phase (11 and 6 days old, respectively) were pretreated by probe sonication to prepare fresh ciliate and cryptophyte lysate for the experiment (Table 1). More specifically, 100 mL of the *M. rubrum* culture (4 × 10^3^ cells/mL) or 50 mL of the *T. amphioxeia* culture (2 × 10^5^ cells/mL) were probe sonified (Scientz JY92-IIN, Ningbo, China) using a repeated pulse cycle (3 s sonify/3 s pause = 90 s of active sonication, power = 400 W) over three minutes to lyse all cells. The duration and amplitude were determined by a preliminary experiment in which time of active sonification was increased from 60 s to 120 s over 200 W to 400 W (data not shown). No intact ciliate or cryptophyte cells were observed under a light microscope at 400 W after 60 s ultrasonic treatment.

Triplicate subsamples of experimental cell lysate were immediately scanned under light microscope using a Sedgewick-Rafter counting chamber to ensure the absence of living or whole cells. Particles of the ciliate lysate were photographed and measured using an Olympus CKX53 inverted microscope (Olympus, Shinjuku, Tokyo) with Infinity Analyze imaging software (Lumenera Corporation, Ottawa, Canada). All particles were evaluated as spheres, and therefore, were fit to a circle and diameter and area measurements collected.

### 5.3. Experimental Design

Triplicate maintenance cultures of *Dinophysis acuminata* and *Mesodinium rubrum* were starved, in the light, for two weeks before the experiment to ensure the prey were fully consumed from the medium and that any responses measured in *Dinophysis* were due to the amendments and not sustained growth or divisions using internal reserves. To begin the experiment, triplicate 1-L flasks were inoculated with *Dinophysis* monoculture at an initial concentration of 150 cell/mL for all seven treatments (Table 1). Live ciliate prey were then provided to *Dinophysis* at two initial concentrations, 1500 and 3000 cells/mL: the Prey_ciliate_^1500^ and Prey_ciliate_^3000^ treatments, respectively. To determine if prey was equally supportive of growth and toxin production when provided as organic matter, half of the ciliates were lysed and then fed to *Dinophysis* as a mixture of live prey and lysate, equivalent to 3000 cells/mL: the Prey + Lysate_ciliate_^3000^ treatment. Additionally, ciliate lysate equivalent to 3000 cells/mL was offered alone to determine if organic “dead” particles could support *Dinophysis* growth and toxin production: the Lysate_ciliate_^3000^ treatment. Prey to predator ratios for all the 1500 cells/mL and 3000 cells/mL ciliate treatments were 10:1 and 20:1, respectively.

Two additional treatments were included to determine if similar growth and toxin production could be achieved by *Dinophysis* through exposure to lysed cryptophytes, Lysate_crypto_^15000^, with an equivalent live cryptophyte treatment run in parallel: Prey_crypto_^15000^ (Table 1). All treatments were compared to a monoculture *Dinophysis* control in which no prey or lysate was provided, and the triplicate flasks were instead supplemented with fresh f/6-Si medium to reach a similar final volume of 500 mL.

Samples, 1.2 mL, were taken every three days from each flask, fixed with 3% (*v*/*v*) formalin solution, and the dinoflagellates, ciliates, and cryptophytes were enumerated microscopically using a Sedgewick-Rafter counting chamber at 100× magnification.

### 5.4. Toxin Analysis

Culture was harvested and separated into medium and cells of *Dinophysis* once experimental cultures reached two growth phases, exponential and plateau. Initial toxin samples were also collected and analyzed from the *Dinophysis* inoculum cultures. Cells were separated from medium using a 15-µm Nitex sieve affixed to polyvinyl chloride (PVC) pipe and back-washed into a pre-weighed 15-mL centrifuge tube (*w*_1_) using fresh media; the sieved medium was collected into a beaker. As such, intracellular (≥15 µm) and extracellular (<15 µm) fractions of each sample were collected, extracted, and analyzed separately. In order to determine the total number of harvested cells for extraction, 200 µL subsamples were pipetted from the intracellular toxin samples into a 2-mL micro-centrifuge tube containing 1.0 mL filtered seawater and 37 µL formalin solution (3% *v*/*v*). The fixed and diluted subsamples were enumerated microscopically to calculate cell concentration using a Sedgewich-Rafter counting chamber at 100× magnification. The 15-mL tube was then reweighed (*w*_2_) to calculate the volume of harvested *Dinophysis* cells (*v*) using the formula:

(1) $$v=\frac{w_{2}-w_{1}}{\rho_{seawater}}$$

where *ρ*_seawater_ was set at 1.03 g/mL given the salinity of the culture medium was 30. The volume harvested (*v*) was then multiplied by the cell concentration to estimate the total number of cells in the tube. Tubes were then frozen at −80 °C for over 24 h before toxin extraction.

Methods of solid phase extraction (SPE) were used as described in [38] to clean up samples prior to analysis. SPE cartridges (Oasis HLB 60 mg; Waters, Milford, MA, USA) were conditioned with 6 mL of methanol and rinsed with 6 mL of Milli-Q water in preparation for cell or media. Culture media (extracellular fraction, <15 µm) was loaded onto the column immediately after separation from cells with no additional processing. Cell samples (intracellular fraction, ≥15 µm), however, underwent a freeze/thaw cycle and bath sonification (KQ-3200E, ultrasonic power = 150 watt, frequency = 40 KHz, Kunshan Ultrasonic Instruments Co., Ltd., Kunshan, China) for 15 min at room temperature, to aid in cell lysis before being loaded onto the conditioned SPE cartridges. The cartridges were then washed with 3 mL of Milli-Q water and toxins were ultimately eluted with 1 mL of methanol into an autosampler vial. Eluates from the samples were heated at 40 °C in a heating block, dried under a stream of high-purity N_2_ (HP-S016SY, ), and re-suspended in 1 mL of methanol for toxin analysis to remove error associated with varying elution volumes. Eluates were frozen at −20 °C until analysis.

The Dionex UltiMate 3000 liquid chromatrography system (Thermo Scientific^TM^ Dionex^TM^, Waltham, MA, USA) coupled with an AB 4000 mass spectrometer (SCIEX, Framingham, MA, USA) with electrospray ionization (LC-MS/MS) was used for the analysis. Toxins, okadaic acid (OA) and dinophysistoxin-1 (DTX1), were analyzed in negative mode, and pectenotoxin-2 (PTX2) in positive mode. Chromatographic separation for OA and DTX1 was performed using a Waters XBridge^TM^ C18 column (3.0 × 150 mm, 3.5 μm particle size) at 40 °C in negative mode. The mobile phase used during negative mode consisted of phase A, 0.05 *v*/*v* % ammonia hydroxide (pH 11) in water and phase B, 0.05 *v*/*v* % ammonia hydroxide in 90% acetonitrile, with a flow rate of 0.4 mL/min and 10 µL injection. A linear gradient elution from 10% to 90% B was run for 9 min, held for 3 min at 90% B, decreased to 10% B in 2 min and held at 10% B for 4 min to equilibrate at initial conditions before the next run was started. In positive mode, Waters XBridge^TM^ C18 column (2.1 × 50 mm, 2.5 μm particle size) at 25 °C was performed for chromatographic separation of PTX2. The mobile phase consisted of phase C, water and phase D, 95% acetonitrile, both contain a constant concentration of buffer (2 mM ammonium formate and 50 mM formic acid). A linear gradient from 10% to 80% acetonitrile was run between 0 and 3 min, held at 80% acetonitrile for 2 min, decreased to 10% in 2 min and held another 2 min. The flow rate during positive mode runs was 0.3 mL/min and the injection volume was set to 10 µL.

The mass spectrometer was operated in multiple reaction monitoring (MRM) mode. Transitions of [M + NH_4_^+^] ion: PTX2, *m*/*z* 876.5 > 823.4, [M-H^+^] ions: OA, *m*/*z* 803.5 > 255.0 and DTX1, *m*/*z* 817.5 > 255.0 were selected for quantitation. The operation conditions were as follows: ion spray voltage (ISV): −4.5 kV, temperature (TEM): 600 °C, nebulizer gas (NEB) 13 psi, curtain gas (CUR): 13 psi, collision gas (CAD): 5 psi in negative mode and ISV: 3 kV, TEM: 650 °C, NEB: 14 psi, CUR: 16 psi, CAD: 5 psi in positive mode. Standards for OA, DTX1, and PTX2 were purchased from the National Research Council, Canada (NRC). Five-point standard curves were generated using NRC reference materials, with concentrations ranging from 1.25 to 20 ng/mL for OA and DTX1, and 6.25 to 100 ng/mL for PTX2.

### 5.5. Calculations

*Dinophysis* growth rate was calculated over the entire period of exponential growth phase (Table 1) using the following formula [55]:

(2) $$\mu =\frac{\ln(N_{2}/N_{1})}{t_{2}-t_{1}}$$

where *N*_1_ and *N*_2_ (cells/mL) are the cell concentrations at time 1 and time 2, respectively. Sampling times are represented by *t*_1_ and *t*_2_ with units of day, and μ is the growth rate calculated at the sampling interval with units of day^−1^.

Toxin data are presented as cellular toxin content or quota (toxin amount per cell), total toxin concentration (intracellular + extracellular, i.e., total toxin in a milliliter of culture), proportion of extracellular toxin (extracellular/ (intracellular + extracellular) × 100%) and net toxin production rate R_tox_ (amount toxin/cell/d). The R_tox_ was calculated using the total toxin concentration (extracellular + intracellular) as described by the authors of [56].

(3) $$R_{tox}=\frac{\left(T_{2}-T_{1}\right)}{\left(\bar{C})(t_{2}-t_{1}\right)}$$

(4) $$\bar{C}=\frac{C_{2}-C_{1}}{\ln(C_{2}/C_{1})}$$

In these equations, *T*_1_ and *T*_2_ are the total toxin concentrations (intracellular + extracellular, i.e., total toxin per milliliter of culture) at time 1 and time 2, respectively. Toxin production rates were calculated for two periods of time: from initial to exponential growth phase, and then from exponential into plateau phase (Table 2). The concentrations of *Dinophysis* cells at time 1 and time 2 are represented as *C*_1_ and *C*_2,_ (cells/mL), respectively.

### 5.6. Statistical Analysis

Repeated Measures ANOVA (SigmaPlot v12.0, Systat Software Inc., London, UK) was used to analyze for differences in DSP and PTX2 toxin content, total toxin concentrations, and differences of toxin production rates between treatments or over time. Shapiro-Wilk test was used to test for normality. T-tests were used to analyze for any differences in growth rate and biomass of *Dinophysis* between treatments. Alpha was set at 0.05 for all analyses.

## Figures and Tables

**Table 1:** Prey, lysate, or a mixture of the two were provided as nourishment during culturing experiments with a *Dinophysis acuminata* isolate after dinoflagellates were starved for two weeks in the light. Treatments included the addition of live prey and/or probe-sonified lysate of the ciliate, *Mesodinium rubrum*, delivered at two initial cell concentrations or equivalents (eq.). The cryptophyte, *Teleaulax amphioxeia*, was also provided in two treatments: live prey or lysate. Treatments were compared to a *Dinophysis* monoculture control where no prey or lysate were added, and instead an equivalent volume was replaced with additional fresh f/6-Si medium. Mean (±standard error) measurements of *Dinophysis* growth rate and biomass are provided.

Treatment ID	Prey/Lysate Species	Prey Initial Conc. (Cells/mL)	Lysate Initial Conc. (Cell eq./mL)	*Dinophysis* Growth ^1^		
				Exponential Growth Rate (/d)	Period of Exponential Growth (d)	Max Biomass (cells/mL)
Prey_ciliate_^3000^	*M. rubrum*	3000	-	0.21 (±0.003)	12	2508 (±162)
Prey_ciliate_^1500^	*M. rubrum*	1500	-	0.16 (±0.007)	15	2252 (±110)
Prey + Lysate_ciliate_^3000^	*M. rubrum*	1500	1500	0.25 (±0.002)	12	3902 (±234)
Lysate_ciliate_^3000^	*M. rubrum*	-	3000	0.12 (±0.004)	3	302 (±8)
Prey_crypto_	*T. amphioxeia*	15,000	-	-	-	170 (±8)
Lysate_crypto_	*T. amphioxeia*	-	15,000	-	-	193 (±7)
Control	none	-	-	-	-	187 (±5)

^1^ Dinophysis initial cell concentration was equal for all 6 treatments and the control, 150 cells/mL. The symbol “-“ indicates zero.

**Table 2:** Mean (±standard error) calculations of *Dinophysis* toxin production rate over two growth phases, exponential (expo.) and plateau (plat.). The duration of days used to represent each growth phase, and therefore calculate net toxin production rate (R_tox_), are included for reference. Statistical differences are indicated by uncommon lowercase letters (Two-Way Repeated Measures ANOVA with alpha set to 0.05, *n* = 3).

Treatment ID	Duration of Each Growth Phase (d)		*Dinophysis* R_tox_ (fg/cell/d)					
	Expo.	Plat.	Initial to Exponential			Exponential to Plateau		
			OA	DTX1	PTX2	OA	DTX1	PTX2
Prey_ciliate_^3000^	10	15	46 (±4) ^a^	56 (±4) ^a^	3747 (±177) ^ab^	40 (±4) ^a^	44 (±2) ^a^	2837 (±241) ^a^
Prey_ciliate_^1500^	16	16	39 (±1) ^a^	37 (±1) ^b^	2723 (±234) ^b^	24 (±1) ^b^	26 (±2) ^b^	920 (±58) ^b^
Prey + Lysate_ciliate_^3000^	10	15	65 (±3) ^b^	63 (±5) ^a^	4171 (±573) ^a^	30 (±1) ^ab^	37 (±4) ^ab^	1662 (±372) ^b^

OA, okadaic acid. DTX1, dinophysistoxin-1. PTX2, pectenotoxin-2.

## References

[1] Hallegraeff G.M. (1993). A review of harmful algal blooms and their apparent global increase. Phycologia.

[2] Reguera B., Riobo P., Rodriguez F., Diaz P.A., Pizarro G., Paz B., Franco J.M., Blanco J. (2014). *Dinophysis* toxins: Causative organisms, distribution and fate in shellfish. Mar. Drugs.

[3] Zhou J., Fritz L. (1994). Okadaic acid antibody localizes to chloroplasts in the DSP-toxin-producing dinoflagellates *Prorocentrum lima* and *Prorocentrum maculosum*. Phycologia.

[4] Marasigan A.N., Sato S., Fukuyo Y., Kodama M. (2001). Accumulation of a high level of diarrhetic shellfish toxins in the green mussel Perna viridis during a bloom of *Dinophysis caudata* and *Dinophysis miles* in Sapian Bay, Panay Island, the Philippines. Fish. Sci..

[5] Windust A.J., Wright J.L.C., McLachlan J.L. (1996). The effects of the diarrhetic shellfish poisoning toxins, okadaic acid and dinophysistoxin-1, on the growth of microalgae. Mar. Biol..

[6] Windust A.J., Quilliam M.A., Wright J.L., McLachlan J.L. (1997). Comparative toxicity of the diarrhetic shellfish poisons, okadaic acid, okadaic acid diol-ester and dinophysistoxin-4, to the diatom *Thalassiosira weissflogii*. Toxicon.

[7] Miles C.O., Wilkins A.L., Munday R., Dines M.H., Hawkes A.D., Briggs L.R., Sandvik M., Jensen D.J., Cooney J.M., Holland P.T. (2004). Isolation of pectenotoxin-2 from *Dinophysis acuta* and its conversion to pectenotoxin-2 seco acid, and preliminary assessment of their acute toxicities. Toxicon.

[8] Ito E., Suzuki T., Oshima Y., Yasumoto T. (2008). Studies of diarrhetic activity on pectenotoxin-6 in the mouse and rat. Toxicon.

[9] (2005). Commission regulation (EC) No. 2074/2005 of the European parliament and of the council of 5 December 2005. Off. J. Eur. Commun..

[10] Park M.G., Kim S., Kim H.S., Myung G., Kang Y.G., Yih W. (2006). First successful culture of the marine dinoflagellate *Dinophysis acuminata*. Aquat. Microb. Ecol..

[11] Yih W., Kim H.S., Jeong H.A., Myung G., Kim Y.G. (2004). Ingestion of cryptophyte cells by the marine photosynthetic ciliate *Mesodinium rubrum*. Aquat. Microb. Ecol..

[12] Takishita K., Koike K., Maruyama T., Ogata T. (2002). Molecular evidence for plastid robbery (Kleptoplastidy) in *Dinophysis*, a dinoflagellate causing diarrhetic shellfish poisoning. Protist.

[13] Hackett J.D., Maranda L., Yoon H.S., Bhattacharya D. (2003). Phylogenetic evidence for the cryptophyte origin of the plastid of *Dinophysis* (Dinophysiales, Dinophyceae). J. Phycol..

[14] Janson S., Granéli E. (2003). Genetic analysis of the psbA gene from single cells indicates a cryptomonad origin of the plastid in *Dinophysis* (Dinophyceae). Phycologia.

[15] Johnson M.D., Oldach D., Delwiche C.F., Stoecker D.K. (2007). Retention of transcriptionally active cryptophyte nuclei by the ciliate *Myrionecta rubra*. Nature.

[16] Kim G.H., Han J.H., Kim B. (2016). Cryptophyte gene regulation in the kleptoplastidic, karyokleptic ciliate *Mesodinium rubrum*. Harmful Algae.

[17] Janson S. (2004). Molecular evidence that plastids in the toxin-producing dinoflagellate genus *Dinophysis* originate from the free-living cryptophyte *Teleaulax amphioxeia*. Environ. Microbiol..

[18] Hackett J.D., Tong M., Kulis D.M., Fux E., Hess P., Bire R., Anderson D.M. (2009). DSP toxin production de novo in cultures of *Dinophysis acuminata* (Dinophyceae) from North America. Harmful Algae.

[19] Kamiyama T., Suzuki T. (2009). Production of dinophysistoxin-1 and pectenotoxin-2 by a culture of *Dinophysis acuminata* (Dinophyceae). Harmful Algae.

[20] Kamiyama T., Nagai S., Suzuki T., Miyamura K. (2010). Effect of temperature on production of okadaic acid, dinophysistoxin-1, and pectenotoxin-2 by *Dinophysis acuminata* in culture experiments. Aquat. Microb. Ecol..

[21] Nagai S., Suzuki T., Nishikawa T., Kamiyama T. (2011). Differences in the production and excretion kinetics of okadaic acid, dinophysistoxin-1, and pectenotoxin-2 between cultures of *Dinophysis acuminata* and *Dinophysis fortii* isolated from western Japan. J. Phycol..

[22] Nielsen L.T., Krock B., Hansen P.J. (2012). Effects of light and food availability on toxin production, growth and photosynthesis in *Dinophysis acuminata*. Mar. Ecol. Prog. Ser..

[23] Mafra L.L., dos Santos Tavares C.P., Schramm M.A. (2014). Diarrheic toxins in field-sampled and cultivated *Dinophysis* spp. cells from southern Brazil. J. Appl. Phycol..

[24] Hattenrath-Lehmann T., Gobler C.J. (2015). The contribution of inorganic and organic nutrients to the growth of a North American isolate of the mixotrophic dinoflagellate, *Dinophysis acuminata*. Limnol. Oceanogr..

[25] Tong M., Smith J.L., Richlen M., Steidinger K.A., Kulis D.M., Fux E., Anderson D.M. (2015). Characterization and comparison of toxin-producing isolates of *Dinophysis acuminata* from New England and Canada. J. Phycol..

[26] Gao H., An X., Liu L., Zhang K., Zheng D., Tong M. (2017). Characterization of *Dinophysis acuminata* from the Yellow Sea, China, and its response to different temperatures and *Mesodinium* prey. Oceanol. Hydrobiol. Stud..

[27] Nishitani G., Nagai S., Sakiyama S., Kamiyama T. (2008). Successful cultivation of the toxic dinoflagellate *Dinophysis caudata* (Dinophyceae). Plankton Benthos Res..

[28] Nishitani G., Nagai S., Takano Y., Sakiyama S., Baba K., Kamiyama T. (2008). Growth characteristics and phylogenetic analysis of the marine dinoflagellate *Dinophysis infundibulus* (Dinophyceae). Aquat. Microb. Ecol..

[29] Nielsen L.T., Krock B., Hansen P.J. (2013). Production and excretion of okadaic acid, pectenotoxin-2 and a novel dinophysistoxin from the DSP-causing marine dinoflagellate *Dinophysis acuta*—Effects of light, food availability and growth phase. Harmful Algae.

[30] Mafra L.L., Nagai S., Uchida H., Tavares C.P., Escobar B.P., Suzuki T. (2016). Harmful effects of *Dinophysis* to the ciliate *Mesodinium rubrum*: Implications for prey capture. Harmful Algae.

[31] Papiol G.G., Beuzenberg V., Selwood A.I., MacKenzie L., Packer M.A. (2016). The use of a mucus trap by *Dinophysis acuta* for the capture of *Mesodinium rubrum* prey under culture conditions. Harmful Algae.

[32] Maestrini S.Y., Berland B.R., Grzebyk D., Spano A.M. (1995). *Dinophysis* spp cells concentrated from nature for experimental purposes, using size fractionation and reverse migration. Aquat. Microb. Ecol..

[33] Sampayo T.J., Smayda T.J., Shimizu Y. (1993). Trying to cultivate *Dinophysis* spp.. Toxic Phytoplankton Blooms in the Sea.

[34] Hattenrath-Lehmann T.K., Marcoval M.A., Mittlesdorf H., Goleski J.A., Wang Z.H., Haynes B., Morton S.L., Gobler C.J. (2015). Nitrogenous nutrients promote the growth and toxicity of *Dinophysis acuminata* during estuarine bloom events. PLoS ONE.

[35] Tong M., Smith J.L., Kulis D.M., Anderson D.M. (2015). Role of dissolved nitrate and phosphate in isolates of *Mesodinium rubrum* and toxin-producing *Dinophysis acuminata*. Aquat. Microb. Ecol..

[36] Seeyave S., Probyn T., Pitcher G., Lucas M., Purdie D. (2009). Nitrogen nutrition in assemblages dominated by *Pseudo*-*nitzschia* spp., *Alexandrium catenella* and *Dinophysis acuminata* off the west coast of South Africa. Mar. Ecol. Prog. Ser..

[37] Hattenrath-Lehmann T.K., Marcoval M.A., Berry D.L., Fire S., Wang Z., Morton S.L., Gobler C.J. (2013). The emergence of *Dinophysis acuminata* blooms and DSP toxins in shellfish in New York waters. Harmful Algae.

[38] Smith J.L., Tong M., Fux E., Anderson D.M. (2012). Toxin production, retention, and extracellular release by *Dinophysis acuminata* during extended plateau phase and culture decline. Harmful Algae.

[39] Kim S., Kang Y.G., Kim H.S., Yih W., Coats D.W., Park M.G. (2008). Growth and grazing responses of the mixotrophic dinoflagellate *Dinophysis acuminata* as functions of light intensity and prey concentration. Aquat. Microb. Ecol..

[40] Riisgaard K., Hansen P.J. (2009). Role of food uptake for photosynthesis, growth and survival of the mixotrophic dinoflagellate *Dinophysis acuminata*. Mar. Ecol. Prog. Ser..

[41] Tong M., Kulis D.M., Fux E., Smith J.L., Hess P., Zhou Q., Anderson D.M. (2011). The effects of growth phase and light intensity on toxin production by *Dinophysis acuminata* from the northeastern United States. Harmful Algae.

[42] Harred L.B., Campbell L. (2014). Predicting harmful algal blooms: A case study with *Dinophysis ovum* in the Gulf of Mexico. J. Plankton Res..

[43] Anderson D.M., Kulis D.M., Doucette G.J., Gallagher J.C., Balech E. (1994). Biogeography of toxic dinoflagellates in the genus *Alexandrium* from the northeastern United-States and Canada. Mar. Biol..

[44] Sagert S., Krause Jensen D., Henriksen P., Rieling T., Schubert H. (2005). Integrated ecological assessment of Danish Baltic Sea coastal areas by means of phytoplankton and macrophytobenthos. Estuar. Coast. Shelf Sci..

[45] Smith J.L., Tong M., Kulis D., Anderson D.M. (2018). Effect of ciliate strain, size, and nutritional content on the growth and toxicity of mixotrophic *Dinophysis acuminata*. Harmful Algae.

[46] Haines K.C., Guillard R.R. (1974). Growth of vitamin b12-requiring marine diatoms in mixed laboratory cultures with vitamin b12-producing marine bacteria. J. Phycol..

[47] Azam F. (1998). Microbial control of oceanic carbon flux: The plot thickens. Science.

[48] Sakami T., Nakahara H., Chinain M., Ishida Y. (1999). Effects of epiphytic bacteria on the growth of the toxic dinoflagellate *Gambierdiscus toxicus* (Dinophyceae). J. Exp. Mar. Biol. Ecol..

[49] Gao H., Hua C., Tong M. (2018). Impact of *Dinophysis acuminata* Feeding *Mesodinium rubrum* on Nutrient Dynamics and Bacterial Composition in a Microcosm. Toxins.

[50] Ojamäe K., Hansen P.J., Lips I. (2016). Mass entrapment and lysis of *Mesodinium rubrum* cells in mucus threads observed in cultures with *Dinophysis*. Harmful Algae.

[51] Nagai S., Nitshitani G., Tomaru Y., Sakiyama S., Kamiyama T. (2008). Predation by the toxic dinoflagellate *Dinophysis fortii* on the ciliate *Myrionecta rubra* and observation of sequestration of ciliate chloroplasts. J. Phycol..

[52] García-Portela M., Reguera B., Sibat M., Altenburger A., Rodríguez F., Hess P. (2018). Metabolomic Profiles of *Dinophysis acuminata* and *Dinophysis acuta* Using Non-Targeted High-Resolution Mass Spectrometry: Effect of Nutritional Status and Prey. Mar. Drugs.

[53] Jiang H., Kulis D.M., Brosnahan M.L., Anderson D.M. (2018). Behavioral and mechanistic characteristics of the predator-prey interaction between the dinoflagellate *Dinophysis acuminata* and the ciliate *Mesodinium rubrum*. Harmful Algae.

[54] Tong M., Zhou Q., David K.M., Jiang T., Qi Y., Donald A.M. (2010). Culture techniques and growth characteristics of *Dinophysis acuminata* and its prey. Chin. J. Oceanol. Limnol..

[55] Guillard R.R.L., Stein J.R. (1973). Division rates. Handbook of Phycological Methods: Culture Methods and Growth Measurements.

[56] Anderson D.M., Kulis D.M., Sullivan J.J., Hall S., Lee C. (1990). Dynamics and physiology of saxitoxin production by the dinoflagellates *Alexandrium* spp.. Mar. Biol..

